# Ellagic acid protects against non-alcoholic fatty liver disease in streptozotocin-diabetic rats by activating AMPK

**DOI:** 10.1080/13880209.2021.1990969

**Published:** 2021-12-06

**Authors:** Jozaa Z. ALTamimi, Ghedeir M. Alshammari, Nora A. AlFaris, Reham I. Alagal, Dalal H. Aljabryn, Norah A. Albekairi, Mahmoud Ahmad Alkhateeb, Mohammed Abdo Yahya

**Affiliations:** a Nutrition and Food Science, Department of Physical Sport Science, Princess Nourah bint Abdulrahman University, Riyadh, Saudi Arabia; b Department of Food Science and Nutrition, College of Food and Agricultural Sciences, King Saud University, Riyadh, Saudi Arabia; c Department of Pharmacology and Toxicology, College of Pharmacy, King Saud University, Riyadh, Saudi Arabia; d Department of Basic Medical Sciences, College of Medicine, King Saud bin Abdulaziz University for Health Sciences (KSAU-HS), Riyadh, Saudi Arabia

**Keywords:** T1DM, NAFLD, polyphenols, oxidative stress, inflammation, lipid metabolism

## Abstract

**Context:**

Ellagic acid (EA) is used in traditional medicine to treated hyperlipidaemia.

**Objective:**

This study examined if AMPK mediates the anti-steatotic effect of ellagic acid (EA) in streptozotocin (STZ)-induced type 1 diabetes mellitus in rats.

**Materials and methods:**

Adult male Wistar rats (130 ± 10 g) were divided into 6 groups (*n* = 8 rats/group) as control, control + EA, control + EA + CC an AMPK inhibitor), T1DM, T1DM + EA, and T1DM + EA + CC. The treatments with EA (50 mg/kg/orally) and CC (200 ng/rat/i.p.) were given the desired groups for 12 weeks, daily.

**Results:**

In T1DM-rats, EA reduced fasting glucose levels (44.8%), increased fasting insulin levels (92.8%), prevented hepatic lipid accumulation, and decreased hepatic and serum levels of total triglycerides (54% & 61%), cholesterol (57% & 48%), and free fatty acids (40% & 37%). It also reduced hepatic levels of ROS (62%), MDA (52%), TNF-α (62%), and IL-6 (57.2%) and the nuclear activity of NF-κB p65 (54%) but increased the nuclear activity of Nrf-2 (4-fold) and levels of GSH (107%) and SOD (87%). Besides, EA reduced downregulated SREBP1 (35%), SREBP2 (34%), ACC-1 (36%), FAS (38%), and HMG-CoAR (49%) but stimulated mRNA levels of PPARα (1.7-fold) and CPT1a (1.8-fold), CPT1b (2.9-fold), and p-AMPK (4-fold). All these events were prevented by the co-administration of CC.

**Discussion and conclusions:**

These findings encourage the use of EA to treat hepatic disorders, and non-alcoholic fatty liver disease (NAFLD). Further *in vivo* and *in vitro* studies are needed to validate its potential in clinical medicine.

## Introduction

Non-alcoholic fatty liver disease (NAFLD) is the most common liver disorder worldwide that results from excessive lipid retention in the liver and encompasses a wide spectrum of hepatic conditions ranging from simple fatty liver to non-alcoholic steatohepatitis, cirrhosis, and hepatocellular carcinoma (Bhatt and Smith 2015). NAFLD is a risk factor for the development of renal, cardiovascular, and cerebrovascular disorders (Hazlehurst et al. 2016). The available data indicate that the global prevalence of NAFLD is about 25% (Younossi et al. 2016). In 2014, the prevalence estimates of NAFLD in men was 10.8% versus 14.9% in women, numbers that are projected to increase to reach 21% in women and 18% in men by 2025 (Araújo et al. 2018). Although NAFLD is mainly common in individuals with obesity, type 2 DM (T2DM), and metabolic syndrome (Met S), NAFLD is also common in patients with T1DM (Hazlehurst et al. 2016; Mohamed et al. 2016; Sviklāne et al. 2018; Tripolino et al. 2019; de Vries et al. 2020). Therefore, early detection and management of NAFLD in T1DM is crucial to prevent hepatic damage and other health co-morbidities.

Yet, the pathogenesis of mechanisms of NAFLD in T1DM is still unclear. Available experimental suggest that hepatic oxidative stress due to increased production of reactive oxygen species (ROS) and concomitant reduction in antioxidants in response to an increasing influx of glucose and free fatty acids (FFAs) is the major mechanism responsible for hepatic inflammation, ischemia/reperfusion (I/R), fibrosis, necrosis, and apoptosis (Ucar et al. 2013; Mohamed et al. 2016; Kitade et al. 2017; Masarone et al. 2018). Besides, independent of oxidative stress, T1DM is associated with several independent pathological mechanisms that stimulate hepatic lipid synthesis steatosis. These include impaired VLDL-c secretion, increased glucose uptake and conversion to fats, abnormally upregulated transcription factors including the carbohydrate-responsive element-binding protein (ChREBP), and the sterol regulatory element-binding protein 1 (SREBP1c) (Bhatt and Smith 2015).

The current investigation was undertaken to provide a better understanding of the pathogenesis of NAFLD in T1DM and T2DM. In this context, the role of the 5′AMP-activated protein kinase (AMPK), an important cellular energy sensor, in the process of hepatic steatosis and NAFLD has become increasingly of interest to many researchers around the world (Smith et al. 2016). AMPK is a key signalling molecule that plays a crucial role in regulating hepatic lipid metabolism. Indeed, AMPK inhibits FAs, triglycerides (TGs), and cholesterol (CHOL) synthesis by downregulating SREBP1 and SREBP2 transcription factors, respectively (Tomita et al. 2005; Yang et al. 2009; Knebel et al. 2012; Quan et al. 2013; Tang et al. 2016; de Souza et al. 2017; Ren et al. 2019). It also stimulates the mitochondria fatty acids (FAs) by activating/upregulating the peroxisome proliferator-activated receptor-alpha transcription factor (PPARα) (de Souza et al. 2017; Ren et al. 2019). Of interest, the hepatic levels and activities of AMPK are significantly depleted in animal models of NAFLD, including those induced by high-fat diet (HFD) and T2DM (Fogarty and Hardie 2010; Kulkarni et al. 2013; Lindholm et al. 2013; Smith et al. 2016; Liou et al. 2019). However, the pharmacological activation of AMPK in these animal models ameliorated hepatic damage and steatosis (Smith et al. 2016; Liou et al. 2019). Despite these findings, the impact of T1DM on the hepatic expression of AMPK concerning NAFLD was not well investigated and poorly described in the literature and needs further investigation.

Ellagic acid is a polyphenol abundant in various fruits, including strawberries, pomegranate, guava, walnuts, almonds, and green tea in the form of hydrolyzable tannins called ellagitannins (Evtyugin et al. 2020). In the intestine, ellagitannins are metabolized to EA, which is metabolized into more absorbable metabolites called urolithin, which may mediate its pharmacological effects (Djedjibegovic et al. 2020). At the clinical and experimental levels, EA possessed several pharmacological health benefits, including acting as an anticarcinogenic, antiviral, anti-inflammatory, antibacterial, antimalarial, antidiabetic, antianxiety, and antiatherogenic molecule (Goswami et al. 2014; Ayhanci et al. 2016; Seo et al. 2016; Polce et al. 2018; Aslan et al. 2020). EA is a potent hepatoprotective, nephroprotective, neuroprotective, and cardioprotective agent due to its well-reported antioxidant, anti-inflammatory, and anti-apoptotic effects (Kannan and Quine 2013; Goswami et al. 2014; Polce et al. 2018, Zhou et al. 2019). Indeed, EA prevented liver damage in several animal models, including, at least, alcohol, carbon tetrachloride (CCl_4_) cisplatin, cyclosporine, paracetamol, and HFD-induced diabetic rats.

Besides, EA ameliorated hyperlipidaemia and prevented hepatic steatosis in obese, HFD, and transgenic animals with T2DM via several mechanisms, including suppressing SREBP1a and activation of PPARα. EA also suppressed TGs and cholesterol synthesis and accumulation in primary human adipocytes (hASC) and human hepatoma Huh7 cells via stimulating FAs oxidation (Okla et al. 2015). EA can activate AMPK in the adipocytes and muscles (Poulose et al. 2011). Metabolites of EA (i.e., urolithin A, C, and D) prevented TGs synthesis and accumulations in the adipocytes and hepatocytes by activating AMPK and concomitant downregulation of suppressing fatty acid synthase (FAS) (Kang et al. 2016). Furthermore, EA prevented cholesterol (CHOL) synthesis *in vivo* in cholesterol-fed rats and *in vitro* by activating AMPK and concomitant suppression of the β-hydroxy β-methylglutaryl-CoA reductase (HMGCoAR) (Lee et al. 2020).

These data suggest that EA may also act in a similar mechanism in the livers of T1DM-induced rats. Therefore, this study was conducted with two aims. First, to investigate the expression pattern and activity of AMPK in the livers of rats with T1DM induced by streptozotocin; second, to examine if the anti-steatosis effect of EA is mediated by activation of AMPK.

## Materials and methods

### Animals

Adult male Wistar rats, 130 ±10 g, 7-weeks-old, were supplied from and maintained in the Experimental Animal Care Centre at King Saud University, Riyadh, Saudi Arabia. The rats were housed in plastic cages (4/cage) in a pathogen-free room and had free access to their diet and drinking water, and were kept under controlled, stable ambient conditions (22 ± 2 °C/12-h light/dark cycle). This study followed the guidelines of Animals in Research: Reporting *In Vivo* Experiments (ARRIVE) (Kilkenny et al. 2010). All procedures, protocols, treatments, sampling, and euthanasia were approved by the Official Review Board at Princess Nourah University, Riyadh, KSA (IRB Number 20-0096), Riyadh, KSA, which follows the guidelines established by the European Communities Council Directive of 24 November 1986, or the National Institute of Health Guide (National Institute of Health Publications No. 80-23, Revised 1978) for the care and use of Laboratory Animals for the experimental procedure.

### DM induction

T1DM was induced in rats using an i.p. single bolus of STZ (65 mg/kg) dissolved in 0.5 M citrate solution (cat Ab142155, Abcam, UK) as described by Wang-Fischer and Garyantes (2018). To prevent death from sudden adverse hypoglycaemia, all rats were orally supplemented with 0.5% glucose. Three days later, plasma glucose levels were measured, and rats with levels higher than 340 mg/dL were considered to have T1DM and included in this study.

### Experimental design

After the establishment of T1DM in all rats (i.e., 3 d after the initial injection), control and diabetic rats were randomly selected and classified into 5 groups (*n* = 8/group) and treated directly as the following: 1) control rats (vehicle-treated): were control rats and treated only with an oral dose of 0.1 M NaOH and intraperitoneal (i.p) injected with 0.1% dimethyl sulfoxide (DMSO) (Cat No. 472301, (Sigma Aldrich, St Louis, MO, USA) diluted in phosphate-buffered saline (PBS/pH = 7.4). 2) Control + EA-treated rats: control rats that were orally administered EA (Cat No. E2250, Sigma Aldrich, St Louis, MO, USA) prepared in 0.1 M NaOH, at a final concentration of 50 mg/kg; 3) Control + EA + dorsomorphin (compound C, CC)-treated rats: control rat treated as in group 2 but received a concomitant i.p. dose of CC, an AMPK inhibitor at a final dose of 200 ng/rats/day. 4) T1DM rats: with pre-established T1DM and received a daily oral dose of 0.1 M NaOH and an i.p bolus of 0.1% DMSO as vehicles; 5) T1DM + EA rats: were rats with pre-established T1DM rats and treated orally with EA (50 mg/kg); 6) T1DM + EA + dorsomorphin (compound C, CC)-treated rats: were rats with pre-established T1DM and received a concomitant orally treatment with EA (50 mg/kg) and i.p injected with CC (i.p.) (200 ng/rat/day).

All treatments were given at 10 a.m. every day for 12 weeks on their regimen schedule. Oral treatments were given by gavage using a special stainless steel feeding cannula.

### Drug preparation and dose selection

As recommended by the supplier and research studies, the solubility of EA is enhanced in alkaline conditions and by dissolving in various concentrations of (0.01, 0.1, and 1 M) NaOH (Rosillo et al. 2011; Zuccari et al. 2020). For this reason, we have prepared EA (10 mg/mL) in 0.1 M NaOH, which showed complete solubility. This has also been prepared following our previous study (ALTamimi, AlFaris, Aljabryn, et al. 2021). Besides, intragastric administration of EA, dissolved in either 0.1 or 0.1 M NaOH, at this dose demonstrated potent testicular and nephroprotective in T1DM-induced rats (ALTamimi, AlFaris, Aljabryn, et al. 2021; ALTamimi, AlFaris, Alshammari, et al. 2021). A similar dose of EA also prevented hepatic oxidative damage and I/R in rats (Polce et al. 2018). Besides, oral administration of EA at a closer dose of 40 mg/kg protected against DN, lowered serum levels of TGs, CHOL, and LDL, and increased serum HDL levels (Ahad et al. 2014). On the other hand, CC was dissolved in DMSO and diluted to 0.1% in phosphate buffer saline (PBS/pH = 7.4). The *in vivo* administration, as well as the dose of CC, were adopted from studies by Hasanvand et al. (2016) and Eid et al. (2021), who demonstrated that the rats are tolerable with this dose, which inhibits AMPK in various tissues, including the brain and the heart. This dose has also been confirmed to inhibit AMPK phosphorylation in our preliminary data by more than 78%. Our preliminary data also showed that individual or combined administration of 0.1 M NaOH and/or 0.1% DMSO has no toxicity or systemic side effects (renal, hepatic, cardiac) as compared to control rats which received normal saline, and therefore were used as vehicles the control or model group.

### Serum and tissue collection

By the end of week 2, all rats were fasted overnight and anesthetized by 1.9 mg/kg ketamine/xylazine hydrochloride solution. Blood samples were directly collected from the heart using cardiac puncture in plain tubes and centrifuged at 11,000 *g* for 10 min. All serum samples were collected in new tubes and stored at −20 °C for further biochemical analysis. Then, all rats were euthanized using the cervical dislocation protocol. The livers were collected on ice and cut into smaller pieces. Parts of the livers were placed in 10% buffered formalin. Other parts were snap-frozen in liquid nitrogen and stored at −80 °C for biochemical and molecular analysis.

### Extraction of hepatic lipids from the freshly collected livers

Parts of the freshly collected livers (*n* = 8/group) were directly used to extract lipid using the methanol: chloroform: normal saline method, as described by Folch et al. (1957). Briefly, parts of the livers 0.25 g were homogenized in 10 mL methanol: chloroform solution (1:2 v/v) for 1 h at 4 °C. The mixture was filtrated, and 2 mL of normal saline was added to the mixture. The mixture was vortexed and then centrifuged for 10 min at 1200 *g*. The lower organic layer containing the dissolved lipids was isolated and evaporated. The collected lipids were dissolved in 0.5 mL isopropanol and used for different lipid quantification.

### Biochemical analysis

Frozen tissues were homogenized in ice-cold PBS (pH =7.4) and centrifuged at a speed of 11,300 *g* at 4 °C for 15 min to obtain the supernatants. The supernatants were stored at −80 °C and used later to measure several biochemical parameters. Serum levels of glucose and insulin were measured using rat colorimetric and ELISA kits (Cat No. 10009582 Cayman Chemical, MI, USA and Ca., No. 589501, Ann Arbor, MI, USA, respectively). Serum levels of alanine aminotransferase (ALT) and aspartate aminotransferase (AST), as well as hepatic levels of malondialdehyde (MDA), superoxide dismutase (SOD), and glutathione (GSH), were measured using rat’ ELISA kits (Cat. No. MBS269614; Cat. No. MBS264975, Cat. No. MBS268427, Cat. No. MBS738685, Cat. No. MBS265966, MyBioSource, CA, USA, respectively). Hepatic levels of ROS were measured using a fluorometric kit (Cat. NO. STA-347; Cell Biolabs, CA, USA). Serum and hepatic levels of TGs, HDL, and LDL-c were determined using rats’ specific assay kits (Cat. No. 10010303, Cayman Chemical, MI, Cat. No. K4436, BioVision, Ca, USA, and Cat. No. 79960, Crystal Chemicals, USA, respectively). Serum and hepatic levels of FFAs were measured using an assay kit (Cat. No. K612, BioVision, USA). All assays were conducted for 8 samples/group as per the manufacturers’ instructions.

### Biochemical measurements in the nuclear fraction

The nuclear fractions of the livers collected from all rats (*n* = 8 rats/group) were prepared using a special commercial kit (Ab113474, Abcam, Cambridge, UK). The nuclear activities Nrf2 and NF-κB p65 were determined using commercial assay kits (Cat# Ab207223, Abcam, Cambridge, UK, and Cat. No. 40596, Active Motif, Tokyo, Japan, respectively, and per the manufacturers’ instructions.

### Quantitative real-time PCR

Primers sequences for the amplification of SREBP1, SREBP2, FAS, β-actin, CPT1a, CPT1b, acetyl Co-A carboxylase (ACC-1), β-hydroxy β-methylglutaryl-CoA reductase (HMGCoAR), and PPARα were designed and purchased from ThermoFisher and are shown in Table 1. Total RNA was extracted and reversed using the TRIzol Reagent (ThromoFisher Scientific, USA) as per the manufacturer’s instructions. First-strand cDNA was synthesised using SuperScript™ VILO™ cDNA Synthesis Kit (Cat. No. 11754050, ThromoFisher Scientific, USA). In brief, 4 µL 5× VILO™ Reaction Mix, 2 µL 10× SuperScript™ Enzyme Mix, 5 µL isolated pure RNA (20 ng), and 9 µL DEPC-treated water were placed in each tube and mixed well. The cDNA conditions were: incubation at 25 °C for 10 min, heating for 1 h at 42 °C, and termination at 85 °C for 5 min. qPCR was carried out in 20 µL reaction mixture containing 2 μL cDNA (500 ng/µL), 0.4 μL of 10 μM each primer (200 nm), 10 μL Ssofast Evergreen Supermix, and 7.2 μL of nuclease-free water. qPCR conditions were: inactivation for 1 cycle at 95 °C (30 sec); denaturation for 5 sec at 95 °C (40 cycles), and annealing at 60 °C for 60 sec (40 cycles), and melting for 30 sec at 95 °C (1 cycle). Control samples contained no template cDNA. Quantification was done using the ΔΔCT method against 18sRNA as the reference gene. All procedures were conducted according to the manufacturer's instructions and were done for 8 samples/groups.

**Table 1.** Primers used in the PCR reaction.

Gene	Primers	GenBank accession #	Product length
SREBP-1c	F:5′-GCA AGG CCA TCG ACT ACA TC-3′	NM_001276707.1	161
	R:5′-TTT CAT GCC CTC CAT AGA CAC-3′		
HMG-CoAR	F:5′TGTTCAAGGGGCGTGCAAAGACAA-3′	NM_013134	202
	R:5′-TCAAGCTGCCTTCTTGGTGCATGT-3′		
PPAR-α	F:5′-TGCGGACTACCAGTACTTAGGG-3′	NM_013196.1	116
	R:5′-GCTGGAGAGAGGGTGTCTGT-3′		
SREBP-2	F:5′-CTGACCACAATGCCGGTAAT-3′	NM_001033694.1	204
	R:5′-CTTGTGCATCTTGGCATCTG-3′		
FAS	F:5′-GCC ATT TCC ATT GCC CTT AGC-3′	NM_017332	273
	R:5′-CTG AGC CAA GCA CCG CAC ACT-3′		
CPT1a	F:5′-TCCGAGGCAGGAGCCCCATC-3′	NM_013200.1	142
	F:5′-TCTCGGTCCAGTTTGCGGCG-3′		
ACC-1α	F:5′-GCT GAA GTG AAC TAC CCC TT-3′	NM_017075.2	200
	R:5′-GAG CCA TGC CTC TAG TAC CT-3′		
β-actin	F:5′-ATC TGG CAC CAC ACC TTC-3	NM_031144	291
	R:5′-AGC CAG GTC CAG ACG CA-3′		

### Western blotting

Parts of the frozen livers (75 mg) (*n* = 8 samples/group) were homogenized in 500 µL radioimmunoassay buffer (RIPA) (Cat. No. 89900, ThermoFisher) containing protease inhibitor cocktail (Cat. NO. 78430, ThermoFisher Scientific). The homogenates were centrifuged at a speed of 11,200 *g* for 10 min at 4 °C) and supernatants containing the protein were separated into new tubes. Protein concentrations in all samples were assessed by an assay kit (Cat. No. 5000002, BioRad, TX, USA) and prepared in the loading dye at a final concentration of 2 µg/µL for each sample. All samples were then boiled for 5 min and separated by the sodium dodecyl sulfate-polyacrylamide gel electrophoresis (SDS-PAGE) at a final concentration of 40 µg/well. All gels were then transferred on nitrocellulose membranes, washed, and blocked by 5% of skimmed milk for 1 h. Then the membranes were washed again and incubated with the mouse monoclonal antibodies primary antibodies against AMPK (Cat. No. 2532), p-AMPK (Thr 172) (Cat. No. 50081), or β-actin (Cat. No. 3700) (Cell Signalling Technology) for 2 h at room temperature with rotation. Then the membranes were washed again and blotted with the secondary horse radish-peroxidase (HRP)-conjugated antibody for another 2 h at room temperature. All washing between the steps was done 3 times each of 10 min with 1× Tris-buffered saline-Tween 20 (TBST) buffer. Antibodies were prepared in the TBST buffer. Bands of the interactions between the primary and 2^nd^ antibodies on each blot were developed using a 5 min incubation with ECL pierce west kit substrate reagents (Cat. No. 32109, ThermoFisher) and were scanned and photographed using the C-Di Git blot scanned and associated software (LI-COR, USA). Expression of both AMPK and p-AMPK were expressed relative to the expression of β-actin.

### Light microscope

Freshly collected livers were placed in 10% buffered formalin. After 24 h, all samples were dehydrated in ascending alcohol concentrations (70–100%). All tissues were then cleared in xylene, embedded in paraffin, and cut by the microtome at a thickness of 3 μm. Then, all the slides were routinely stained with haematoxylin and eosin (H&E) or Masson trichrome stain (for fibrosis) and examined under a light microscope by a pathologist who is unaware of the experimental groups. For each slide, 5 fields from different areas were captured.

### Statistical analysis

Statistical analyses for all measured parameters were processed using Graph Pad Prism statistical software package (version 6). Data were analyzed using the 1-way ANOVA followed by Tukey's t-test as a *post hoc* test. Data were considered significantly different at *p* < 0.05 and were presented as means pulse/minus standard deviation (mean ± SD).

## Results

### EA exerts a hypoglycaemic, hypolipidemic, and insulin-releasing effect in an AMPK-dependent manner

Administration of EA to control alone or in conjugation with CC, an AMPK inhibitor, didn’t significantly alter food intake, final body weights, liver weights, nor fasting insulin levels as compared to control rats (Table 2). However, administration of EA to control rats significantly reduced fasting serum insulin, TGs, CHOL, LDL-c, and FFAs levels, as well as hepatic levels of TGs, CHOL, and FFAs as compared to control rats (Tables 2 and 3). No significant alterations in the levels of serum glucose, TGs, CHOL, LDL-c, and FFAs, as well as in the hepatic levels of CHOL, TGs, and FFAs were seen when control + EA + CC-treated rats were compared with the control rats (Tables 2 and 3). Nonetheless, T1DM-induced rats showed a significant reduction in final body and fasting insulin levels with a concomitant increase in the weekly food intake, liver weights, and fasting glucose levels (Table 2). They also showed a significant increase in the serum and hepatic levels TGs, CHOL, and FFAs, as well as in serum LDL levels as compared to control rats (Table 3). All these events were significantly reversed in T1DM + EA as compared to T1DM-induced rats and were prevented by co-admiration of CC (Tables 2 and 3). Except for final body weights, food intake, and liver weights, the levels of all the serum and hepatic endpoints were slightly but significantly higher in T1DM + EA-treated rats as compared to control rats (Tables 2 and 3). However, the levels of all these parameters were not significantly different when T1DM + EA + CC-treated rats were compared with T1DM-induced rats (Tables 2 and 3).

**Table 2.** Alteration in final body weights, food intake, liver weights, and fasting plasma and levels glucose in all experimental groups.

Parameter	Control	Control + EA	Control + EA + CC	T1DM	T2DM + EA	T1DM + EA + CC
Plasma						
Final body weight (g)	412 ± 46	429 ± 31	444 ± 29	339 ± 41^abc^	409 ± 21^d^	319 ± 29^abce^
Liver weights (g)	15.6 ± 2.8	15.9 ± 2.2	14.3 ± 2.5	21.8 ± 3.1^abc^	16.5 ± 2.9^d^	19.9 ± 3.3^abce^
Weekly food intake (g/6 rats)	1212 ± 100	1190 ± 119	1231 ± 87	1876 ± 147^abc^	1321 ± 189^d^	1992 ± 214^abce^
Fasting glucose (mg/dl)	103 ± 7.8	86 ± 6.9^a^	112 ± 6.4^b^	328 ± 22.4^abc^	181 ± 14.5^abcd^	356 ± 33.1^abce^
Fasting insulin	4.6 ± 0.55	4.1 ± 0.67	4.7 ± 0.71	1.4 ± 0.25^abc^	2.7 ± 0.61^abcd^	1.1 ± 0.31^abce^
Serum						
ALT (U/l)	41 ± 7.8	45 ± 8.1	41 ± 5.9	113 ± 11.7^abc^	56 ± 5.7^abcd^	109 ± 8.5^abce^
AST (U/l)	23.4 ± 4.7	25.7 ± 6.1	21.5 ± 6.3	93.5 ± 8.4^abc^	37.1 ± 6.2^abcd^	93.2 ± 7.1^abce^

Data are presented as the mean ± SD for *n* = 8 rats/group. Significance was determined at *p* < 0.05. ^a^: significantly different as compared to control; ^b^: significantly different as compared to control + Ellagic acid (EA); ^c^: significantly different as compared to control + EA + compound C (CC, and AMKP inhibitor); ^d^: significantly different as compared to T1DM-induced rats; and ^e^: significantly different as compared to T1DM + EA-treated rats.

**Table 3.** Changes in liver weight and serum and hepatic fractions in all groups of rats.

Parameter	STD	Control + EA	Control + EA + CC	T1DM	T2DM + EA	T1DM + EA + CC
Serum						
TGs (mg/dl)	46.2 ± 6.7	35.2 ± 4.1^a^	49.8 ± 7.1^b^	121.1 ± 9.8^abc^	61.4 ± 6.3^abcd^	129 ± 8.1^abce^
CHOL (mg/dl)	78.3 ± 7.8	61.2 ± 7.1^a^	83.5 ± 9.1^b^	173 ± 15.4^abc^	73.4 ± 9.2^bd^	181 ± 13.9^abce^
LDL-c (mg/dl)	36.5 ± 5.6	22.6 ± 4.7^a^	40.3 ± 7.5^b^	83.5 ± 7.4^abc^	43.5 ± 6.9^abd^	88.3 ± 8.5^abce^
FFAs (µmol/l)	135 ± 15.4	110 ± 13.4^a^	143 ± 17.4^b^	372 ± 24.5^abc^	222 ± 12.6^abcd^	355 ± 31.3^abce^
Liver						
Triglycerides (µg/g)	724 ± 88.9	609 ± 48.4^a^	812 ± 73.4^b^	3823 ± 413^abc^	1092 ± 112^abcd^	3293 ± 318^abce^
CHOL (µg/g)	218 ± 18.6	178 ± 14.4^a^	231 ± 22.1^b^	518 ± 49.2^abc^	265 ± 34.2^abcd^	539 ± 41.5^abce^
FFA (µmol/g)	43.2 ± 6.8	33.2 ± 4.4^a^	49.3 ± 7.3^b^	132 ± 18.4^ab^	83.3 ± 8.8^abcd^	147 ± 15.6^abce^

Data are presented as the mean ± SD for *n* = 8 rats/group. Significance was determined at *p* < 0.05. ^a^: significantly different as compared to control; ^b^: significantly different as compared to control + Ellagic acid (EA); ^c^: significantly different as compared to control + EA + compound C (CC, and AMKP inhibitor); ^d^: significantly different as compared to T1DM-induced rats; and ^e^: significantly different as compared to T1DM + EA-treated rats.

### EA prevents hepatic lipid accumulation at the histological levels in an AMPK-dependent manner

**Figure 1.**
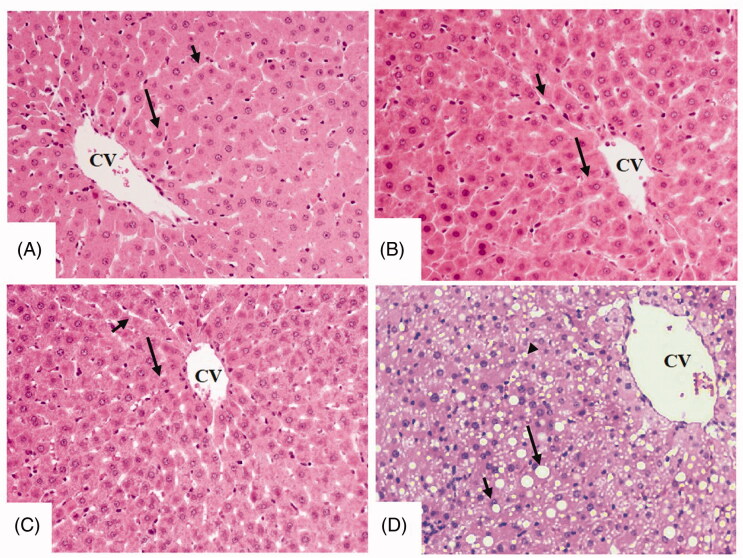
Histological sections from some experimental groups of rats as stained by haematoxylin and eosin (H &E). A, B, and C were taken from control, control + ellagic acid (EA), and control + EA + compound C (CC/AMPK inhibitor), respectively and showing normal liver architectures with rounded normally-appeared hepatocytes (long arrow) radiating from the central vein (CV) with intact sinusoids (short arrow). D was taken from an STZ-diabetic rat (T1DM-induced rat) and showed severe cytoplasmic accumulation of lipid vacuoles of different sizes including large (long arrow), medium (short arrow), and small (arrowhead) vacuoles. 200×.

**Figure 2.**
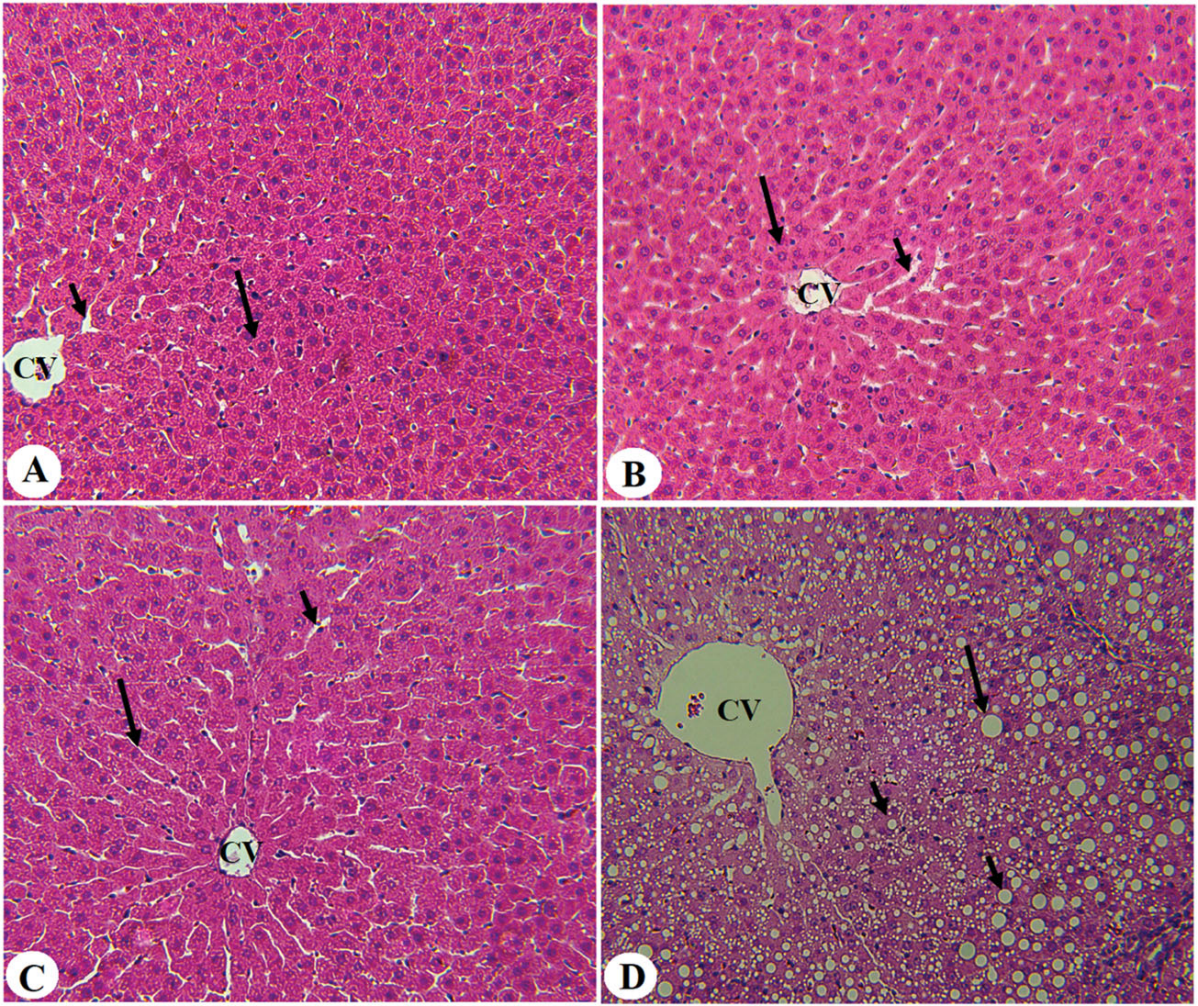
Histological sections from some experimental groups of rats as stained by haematoxylin and eosin (H &E). A was taken for T1DM-induced rats and showed severe congestion in the portal vein (PV) that is surrounded by increased infiltration of inflammatory cells (curved arrow). B was taken from a T1DM + EA-treated rat and showed almost normal architectures like those seen in the control rats with normal hepatocytes (long arrow). However, very few cytoplasmic lipid accumulations are still seen in few hepatocytes. C and D were taken from T1DM + EA + CC and showed similar abnormal features like those observed in T1DM-induced rats including increased accumulation of large, medium, and small cytoplasmic lipid droplets (long arrow, short arrow, and arrowheads, respectively), with increased accumulation of inflammatory cells around the congestive portal vein (PV) (curved arrow).

Livers from control, control + EA, and control + EA + CC-treated rats showed normal histological structures with intact sinusoids and normally appeared hepatocytes radiating from the central vein (Figure 1(A–C)). However, dilated central vein with increased lipid droplets accumulation of all sizes with a parallel increase in inflammatory cells around the portal vein was observed in the T1DM-induced rats (Figures 1(D) and 2(A)). On the other hand, much improvement with almost normal liver structure and very few fat vacuolizations in a limited number of cells were observed in T1DM + EA-treated rats (Figure 2(B)). Similar morphological alterations with increased lipid droplet accumulation and increased inflammatory cell infiltration around the congestive portal vein were observed in T1DM + EA + CC-treated rats (Figure 2(C,D)).

### EA, in an AMPK-dependent manner, suppresses oxidative stress and inflammation by inhibiting NF-κB p65 and activating Nrf2

**Figure 3.**
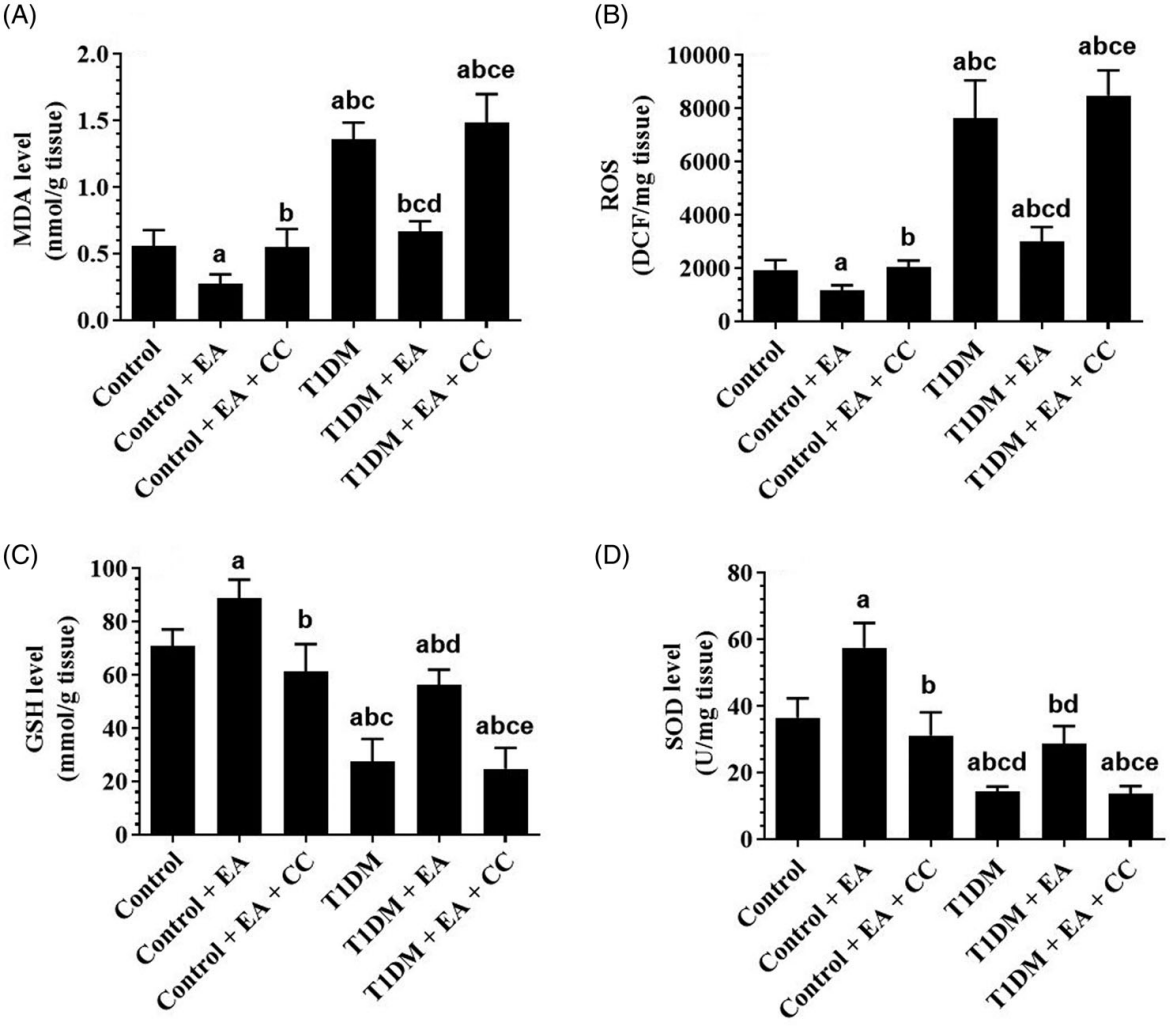
Levels of oxidative stress markers in the livers of all groups of rats. Data are presented as the mean ± SD for *n* = 8 rats/group. Significance was determined at *p* < 0.05. ^a^: significantly different as compared to control; ^b^: significantly different as compared to control + ellagic acid (EA); ^c^: significantly different as compared to control + EA + compound C (CC, and AMKP inhibitor); ^d^: significantly different as compared to T1DM-induced rats; and ^e^: significantly different as compared to T1DM + EA-treated rats.

**Figure 4.**
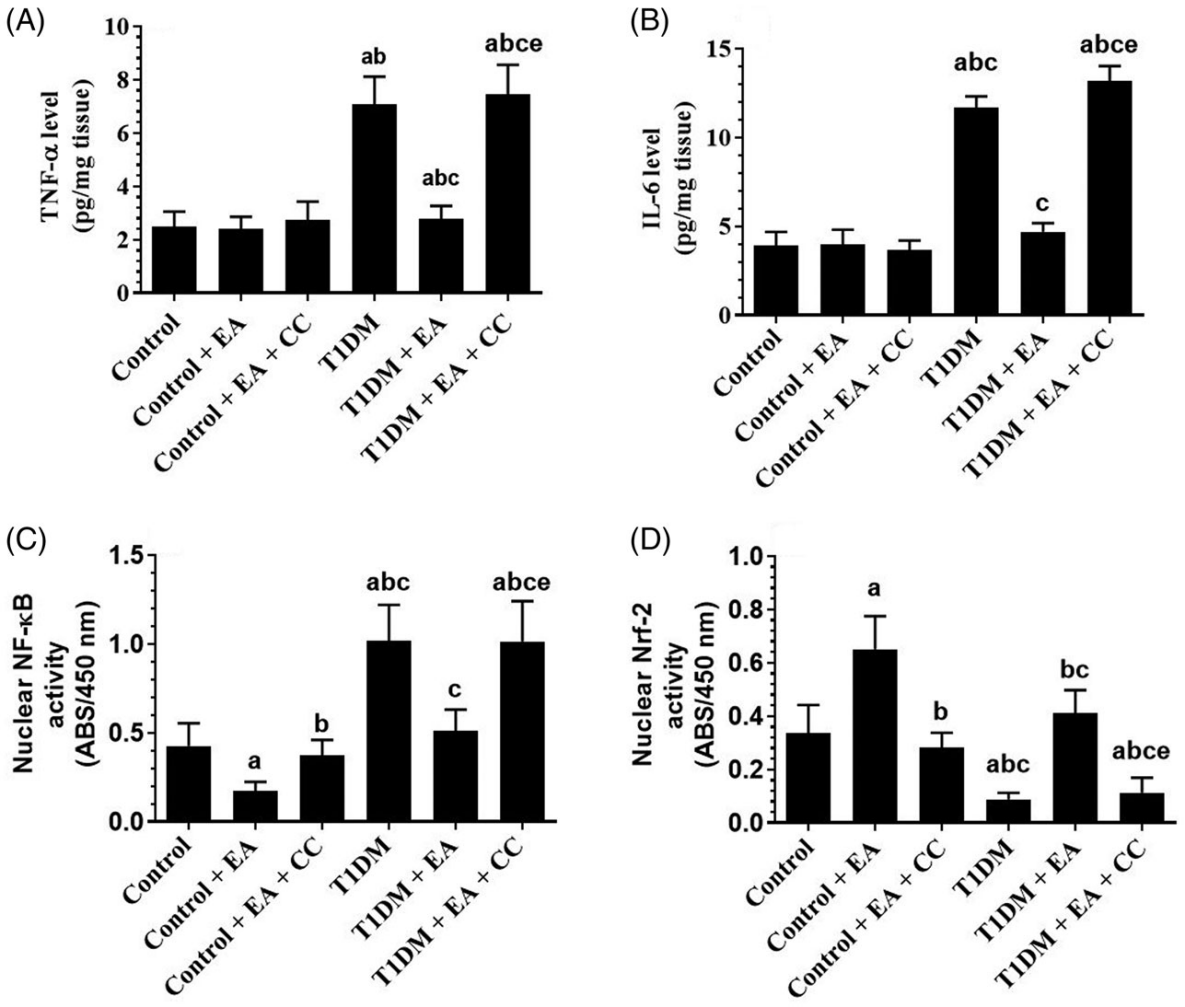
Levels of inflammatory markers and nuclear activities of NF-κB and Nrf2 in the livers of all groups of rats. Data are presented as the mean ± SD for *n* = 8 rats/group. Significance was determined at *p* < 0.05. ^a^: significantly different as compared to control; ^b^: significantly different as compared to control + ellagic acid (EA); ^c^: significantly different as compared to control + EA + compound C (CC, and AMKP inhibitor); ^d^: significantly different as compared to T1DM-induced rats; and ^e^: significantly different as compared to T1DM + EA-treated rats.

Levels of ROS, MDA, TNF-α, IL-6, as well as the nuclear activity of NF-κB p65, were significantly increased but the nuclear activity of Nrf2 was significantly decreased in the liver of T1DM-induced rats as compared to control rats (Figures 3(A–D) and 4 (A–D)). However, hepatic levels of TNFα and IL-6, levels of ROS, MDA, and nuclear levels of NF-κB p65 were significantly decreased and the nuclear levels of Nrf-2 were significantly increased in the levels of both the control + EA and T1DM + EA-treated rats as compared either to the control or T1DM-induced rats, respectively (Figures 3(A–D) and 4(A–D)). Also, hepatic levels of TNF and IL-6 were not significantly different between the control and control + EA-treated rats but were significantly decreased in the livers of T1DM + EA-treated rats as compared to T1DM-induced rats (Figure 4(A,B)). No significant variation in the levels of all these markers was seen when control + EA + CC were compared to control rats or when T1DM + EA + CC were compared with T1DM-induced rats (Figures 3(A–D) and 4(A–D)).

### EA stimulates the phosphorylation of AMPK in the livers of both the control and T1DM-induced rats

**Figure 5.**
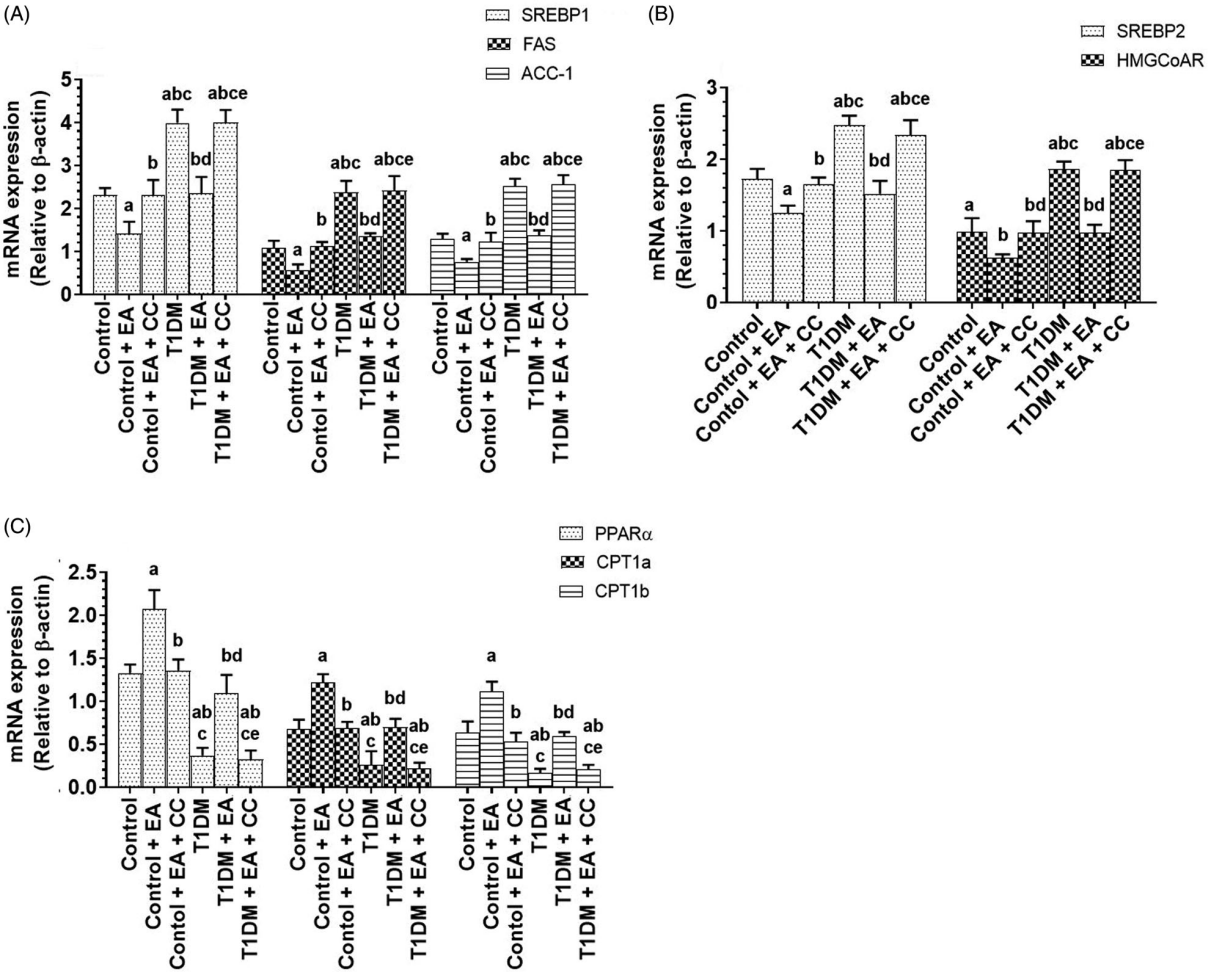
mRNA of lipogenic and fatty acid oxidation-related genes the livers of all groups of rats. Data are presented as the mean ± SD for *n* = 8 rats/group. Significance was determined at *p* < 0.05. ^a^: significantly different as compared to control; ^b^: significantly different as compared to control + ellagic acid (EA); ^c^: significantly different as compared to control + EA + compound C (CC, and AMKP inhibitor); ^d^: significantly different as compared to T1DM-induced rats; and ^e^: significantly different as compared to T1DM + EA-treated rats.

Total protein levels of AMPK were not significantly varied among all the experimental groups (Figures 5(A)). Protein levels of p-AMPK (Thr^172^) were significantly decreased in the livers of T1DM-induced rats as compared to control rats (Figure 5(A)). The protein levels of p-AMPK (Thr^172^) were significantly increased in the livers of the control + EA as compared to control rats and in the livers of T1DM + EA-treated rats as compared to T1DM-induced rats (Figure 5(A)). The protein levels of p-AMPK (Thr^172^) in the livers T1DM + EA were not significantly different from those measured in the livers of the control rats (Figure 5(A)). However, protein levels of p-AMPK were significantly decreased in both the control + EA + CC and T1DM + EA + CC as compared to their levels depicted in the livers of the control + EA-treated rats or T1DM + EA-treated rats, respectively (Figure 5(A)). No, significant variations in the protein levels of p-AMPK were seen when control + EA + CC was compared with control rats or when T1DM + EA + CC-treated rats were compared with T1DM-induced rats (Figure 5(A)).

### EA suppresses hepatic lipogenesis by suppressing SREBP1/2 and activating the PPARα/CPT1, in an AMPK-dependent manner

**Figure 6.**
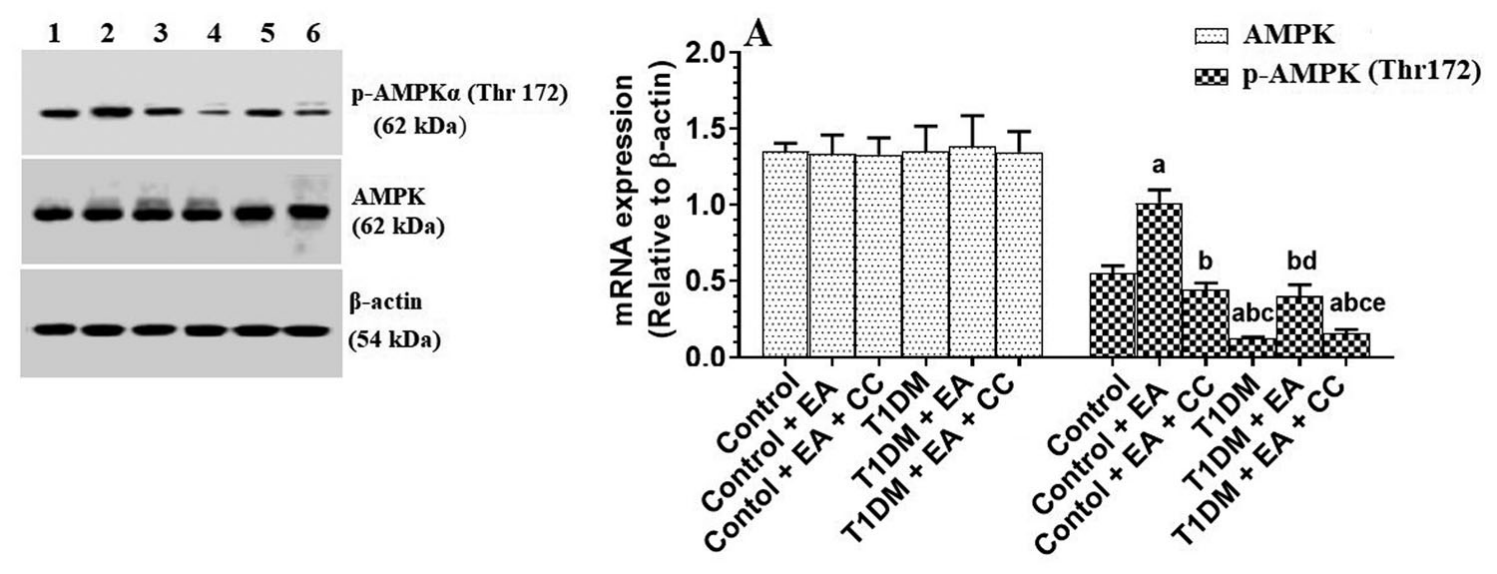
Total and phosphorylated levels of AMPK in the livers of all groups of rats. Data are presented as the mean ± SD for *n* = 8 rats/group. Significance was determined at *p* < 0.05. ^a^: significantly different as compared to control; ^b^: significantly different as compared to control + ellagic acid (EA); ^c^: significantly different as compared to control + EA + compound C (CC, and AMKP inhibitor); ^d^: significantly different as compared to T1DM-induced rats; and ^e^: significantly different as compared to T1DM + EA-treated rats.

mRNA levels of SREBP1, SREBP2, FAS, HMGCoAR were significantly increased but mRNA levels of PPARα, CPT1a, and CPT1b were significantly decreased in the livers of T1DM-induced rats as compared to control rats (Figure 6(A–C)). mRNA levels of SREBP1, SREBP2, FAS, HMGCoAR were significantly decreased but mRNA levels of PPARα, CPT1a, and CPT1b were significantly increased in the livers of both control + EA and T1DM-treated rats as compared to either the control rats or T1DM-induced rats, respectively (Figure 6(A–C)). mRNA levels of all these proteins were not significantly different between T1DM + EA-treated rats as compared to control rats (Figure 6(A–C)). However, administration of CC to both the control + EA or T1DM + EA significantly increased mRNA levels SREBP1, SREBP2, FAS, HMGCoAR and decreased mRNA levels of PPARα, CPT1a, and CPT1b as compared to either the control + EA-treated rats or T1DM + EA-treated rats, respectively (Figure 6(A–C)). No significant variation in mRNA levels of all these genes was seen when a comparison was made between the control and control + EA + CC or between T1DM + EA + CC and T1DM-induced rats (Figure 6(A–C)).

## Discussion

**Figure 7.**
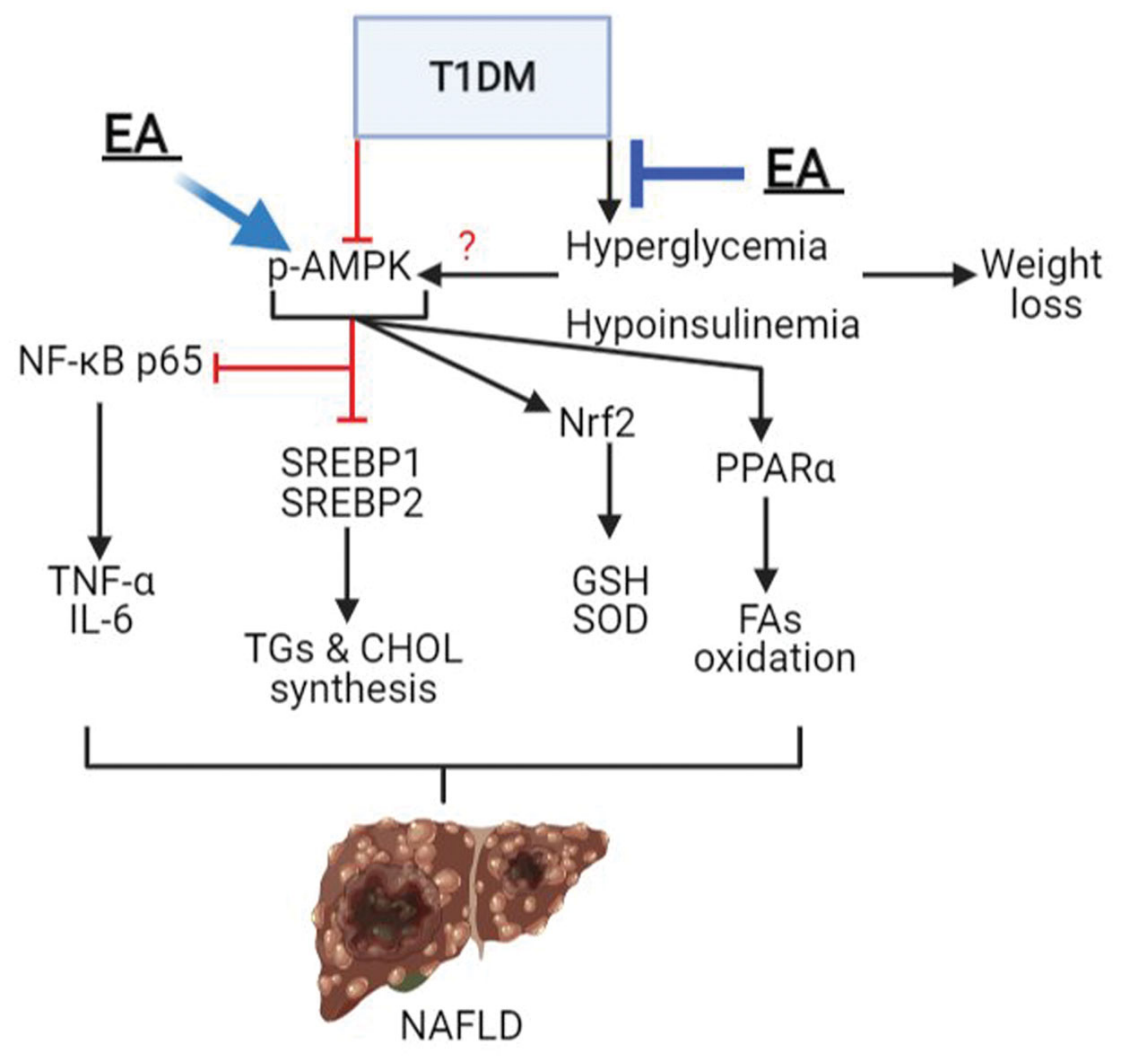
A graphical summary demonstrating the possible mechanism of protection afforded by ellagic acid (EA) against type 1 diabetes mellitus (T1DM)-induced liver damage and hepatic steatosis. In the liver, AMPK inhibits cellular inflammation and cytokine synthesis (i.e., tumour necrosis factor-α (TNF-α) and interleukin-6 (IL-6)) by inhibiting the transcription factor, NF-κB. Also, AMPK inhibits triglycerides (TGs) and cholesterol (CHOL) synthesis by suppressing the transcription factors SREBP1 and SREBP2, respectively, as well as increasing fatty acid synthesis mediated by activating PPARα. In addition, AMPK stimulates the cellular antioxidant system by activating Nrf2 and subsequent increases in the levels of glutathione (GSH) and superoxide dismutase (SOD). T1DM-induced hyperglycaemia and hypoinsulinemia are associated with a significant reduction in hepatic AMPK activities (p-AMPK) and increased risk for the development of non-alcoholic fatty liver disease (NAFLD) due to increased hepatic oxidative stress, inflammation, and lipid accumulation. EA prevents T1DM-induced liver damage and the progression to NAFLD by its hypoglycaemic effect and its ability to stimulate hepatic AMPK activity.

The findings of this study show that chronic administration of EA ameliorates hepatic damage and steatosis in STZ-induced T1DM in rats, mainly by its hypoglycaemic and antioxidant effects and activating AMPK. Suppression of AMPK by CC abolished all these beneficial health benefits of EA effects. A summary of these findings is summarized in Figure 7.

T1DM is characterized by sustained hyperglycaemia, hypoinsulinemia. Polyphagia and weight loss is a common feature of T1DM due to increased muscle wasting and peripheral lipolysis in response to the severe reduction in insulin levels (Enoksson et al. 2003). We have validated our animal model by the obvious increase in fasting glucose and insulin levels, as well as the higher food intake and the significant reduction in rats’ body weights by the end of the study. Besides, T1DM-induced rats had significantly higher serum and hepatic levels of FFAs, which confirmed a higher peripheral lipolysis rate. All these effects were ameliorated by the co-administration of EA, thus suggesting potent hypoglycaemic and antidiabetic properties of EA against T1DM. In particular, EA lowered fasting blood glucose levels in both control and STZ-diabetic rats but stimulated insulin levels and restored normal body weights in the STZ-diabetic rats. In the same line, the hypoglycaemic and insulin lowering potential of EA was reported in rodents with T1DM and T2DM and was mediated by several mechanisms including hepatic stimulating β-survival, and ameliorating peripheral insulin resistance (Fatima et al. 2017; Nankar and Doble 2017; Polce et al. 2018; Farbood et al. 2019). However, our data extend these findings and add a possible mechanism linking all these effects together.

Accordingly, we have found that the hypoglycaemic but not the insulin stimulatory effect of EA is AMPK-dependent. Activities of AMPK were significantly reduced in the livers of STZ-diabetic rats of this study and were significantly increased by the administration of EA. Supporting our data, AMPK levels are significantly reduced in rodents with NAFLD (Fogarty and Hardie 2010; Lindholm et al. 2013; Smith et al. 2016; Liou et al. 2019; Zhang et al. 2019). However, we have found that the concurrent administration of CC along with EA abolished its hypoglycaemic effect. Indeed, AMPK can lower fasting blood glucose levels by suppressing hepatic gluconeogenesis, improving hepatic and peripheral insulin sensitivity, and stimulate GLUT-4 expression on the muscle cells (Viollet et al. 2009; Ruderman et al. 2013). Also, EA and its metabolites urolithin A, C, and D, stimulated AMPK activity in primary adipocytes, hepatoma Huh7 cells, and livers of high-CHOL-fed rats (Poulose et al. 2011; Kang et al. 2016; Lee et al. 2020).

On the other hand, hyperglycaemia stimulates the generation of ROS in the livers of diabetic rats by increasing influx of FFAs and inflammatory cytokines from the impaired adipose tissue, damaging the mitochondria, and activation of several ROS-generating pathways (Ghosh et al. 2015; Mohamed et al. 2016; Lee et al. 2017). In diabetic rats of both types, ROS not only irreversibly induces oxidative modification to protein, lipids, and DNA, but also increase the infiltration of leukocytes, activate inflammation by regulating NF-κB, and induces cell apoptosis mainly by activating the intrinsic (mitochondria-mediated) cell death (Panasiuk et al. 2006; Baffy 2009; Francés et al. 2010; Palsamy et al. 2010; Romagnoli et al. 2010; Leung and Nieto 2013; Ghosh et al. 2015; Rodríguez et al. 2018; Kawai et al. 2020; Lee et al. 2020). However, levels of Nrf2 and its downstream cellular antioxidants, including GSH and other antioxidant enzymes, are significantly depleted under hyperglycaemic conditions and participate significantly in T1DM-induced liver damage (Ahmed 2005; Han et al. 2006; Parveen et al. 2010; Elango et al. 2016).

Higher levels of ROS and MDA (a marker of lipid peroxidation), reduced levels of GSH and SOD, increased nuclear activation of NF-κB p65, higher levels of TNF-α, and IL-6 were observed in the livers of STD-diabetic rats. However, EA was able to reverse all these events in the livers of diabetic rats. Although these events could be explained by ameliorating hyperglycaemia and improving insulin levels, EA also reduced levels of ROS and MDA and the nuclear activation of NF-κB and stimulated the nuclear activity and levels of GSH and SOD in the livers of control rats. These data suggest potent antioxidant, anti-inflammatory, and anti-apoptotic effects of EA, possibly through the transactivation of Nrf2.

Supporting these data, the hepatic protective effect of EA has been also confirmed in alcohol, ConA, Hg, CCl_4_, paracetamol, cisplatin, rifampicin, CsA, and D-Gal/LPS-induced liver damage where the protection was mediated by inhibiting ROS generation and activating Nrf2/antioxidants axis (reviewed in García-Niño and Zazueta (2015). In this review, the unique structure of EA, which contains four hydroxyls and two lactone functional groups, can scavenge different types of free radicals (Cozzi et al. 1995; Nugroho et al. 2014). EA prevented liver, kidney, lung, and keratinocyte damage and apoptosis by activating Nrf2 and stimulating GSH and antioxidant enzymes (Barch and Rundhaugen 1994; Shepherd et al. 2000; Hseu et al. 2012; Celik et al. 2013; Kim et al. 2013; Gu et al. 2014; Vijaya Padma et al. 2014). Also, EA inhibited NF-κB in the renal tissues of T1DM (Ahad et al. 2014). In the same way, EA protected the mice against sleep deprivation-induced memory loss by suppressing TLR-4/NF-κB and activating Nrf2 (Wang et al. 2021). EA prevents hepatic fibrosis in several animal models by downregulating TGF-β1 expression and activating several fibrinolytic molecules such as matrix metalloproteinases-2/9 (MMP-2 and MMP-9) (Devipriya et al. 2007; Suzuki et al. 2009).

Another interesting observation noticed in this study is that the effects of EA on the activities of both NRf2 and NF-κB in the livers of both the control and STZ-diabetic rats were largely abolished by suppressing AMPK with CC, thus confirming that the antioxidant and anti-inflammatory effects of EA are AMPK dependent. Indeed, several lines of evidence have shown that AMPK can decrease oxidative stress and inflammation in various stress conditions by downregulating the transcription of NF-κB, suppressing NF-κB signalling and inflammatory cytokines synthesis, and stimulating Nrf2 expression and nuclear activation (Peairs et al. 2009; Salminen et al. 2011; Wang et al. 2012; Sid et al. 2013; Mo et al. 2014; Zimmermann et al. 2015; Xu et al. 2018; Xiang et al. 2019).

NAFLD is a common feature observed in both T1DM and T2DM (Bhatt and Smith 2015; Hazlehurst et al. 2016). The disease is characterized by several biochemical and histological alterations due to different mechanisms including hyperglycaemia, increase the influx of FFAs from adipose tissue, hepatic I/R, and oxidative damage (Mohamed et al. 2016; Sviklāne et al. 2018; Tripolino et al. 2019). The initial stage of the disease includes an accumulation of fat vacuoles in the cytoplasm of the hepatocytes (microvascular steatosis) which progressive in the later stages to macrovesicular steatosis, where the lipid vacuole reside in the nuclei (Tripolino et al. 2019). In the presence of increased inflammatory cell infiltrates and pericellular, perisinusoidal, and periportal fibrosis, NAFLD can develop to steatohepatitis (Tripolino et al. 2019). These conditions are associated with dyslipidemia and significant increment in the circulatory levels of liver injury-related enzymes. Similar observations were also seen in the livers of STZ-induced rats of this study, thus confirming previously reported studies that T1DM in both humans and experimental animals are associated with NAFLD and dyslipidemia (Hazlehurst et al. 2016; Mohamed et al. 2016; Sviklāne et al. 2018; Tripolino et al. 2019; de Vries et al. 2020). However, the ability of EA to reduce hepatic lipid accumulation, ameliorate the resulted dyslipidemia, and the increment in the levels of ALT and AST with the previously discussed ameliorative effect on liver fibrosis was the clearest evidence that EA could prevent NAFLD in T1DM-induced rats.

Stimulated lipogenesis mediated by overexpressing SREBP1 and SREBP2 and their target genes (i.e., FAS/ACC-1 and HMGCoAR) with a concomitant reduction in β-oxidation due to suppression of PPARα/CPT1 axis mediated NAFLD in diabetic animals (Oliveira et al. 2012) and were shown in this study. On the contrary, chronic administration of EA suppressed the transcription of SREBP1, SREBP2, FAS, ACC-1, and HMGCoAR and significantly upregulated levels of PPARα and CPT1a, and CPT1b not in only in the livers of T1DM-induced rats but also in the livers of control rats. Although these data are being unique to be shown in the T1DM-induced rats of this study, many previous studies have shown a protective effect of EA against NAFLD in rodents with T2DM (Yoshimura et al. 2013; García-Niño and Zazueta 2015; Okla et al. 2015; Kang et al. 2016; Zhang et al. 2019; Lee et al. 2020).

The current study demonstrates that EA also ameliorates hepatic steatosis by activating AMPK. This was confirmed later where CC prevented the anti-steatotic effect of EA and its impact on these transcription factors in the livers of both the control and STZ-diabetic rats. Indeed, AMPK is a novel molecule that can treat NAFLD and NASH by inhibiting lipogenesis through suppressing SREBP1/2 and upregulating PPARα (Strzyz 2020; Tian et al. 2020). Therefore, we could strongly suggest that EA prevents hepatic steatosis in STZ-induced T1DM in rats by activating hepatic AMPK.

## Conclusions

The data reported in this study confirm the ability of EA to treat NAFLD in diabetic animals and show a possible mechanism of action. Therefore, these data encourage further subclinical and clinical trials, which could provide a novel therapy to alleviate the complication associated with this disorder in affected individuals.
